# Subcutaneous Administration of PDGF-AA Improves the Functional Recovery After Spinal Cord Injury

**DOI:** 10.3389/fnins.2019.00006

**Published:** 2019-01-22

**Authors:** Xue-Yan Guo, Fei-Xiang Duan, Jing Chen, Ying Wang, Rui Wang, Lin Shen, Qi Qi, Zhi-Quan Jiang, An-You Zhu, Jin Xi, He-Zuo Lü, Jian-Guo Hu

**Affiliations:** ^1^Department of Clinical Laboratory, The First Affiliated Hospital of Bengbu Medical College, Bengbu, China; ^2^Anhui Key Laboratory of Tissue Transplantation, Bengbu Medical College, Bengbu, China

**Keywords:** platelet-derived growth factor-AA, subcutaneous administration, spinal cord injury, functional recovery, oligodendrocytes

## Abstract

Previous studies by our group have demonstrated that the transplantation of exogenous platelet-derived growth factor (PDGF)-AA-overexpressing oligodendrocyte progenitor cells (OPCs) promotes tissue repair and recovery of neurological function in a rat model of spinal cord injury (SCI). However, it remains unclear whether treatment with PDGF-AA also affects endogenous oligodendrocytes (OLs) or even neurons, thus promoting further functional recovery after SCI. In the present study, we evaluated the therapeutic potential of PDGF-AA treatment by direct subcutaneous injection of PDGF-AA immediately after SCI. We demonstrated that PDGF-AA injection resulted in increased tissue sparing, myelination and functional recovery in rats following SCI. Further experimentation confirmed that PDGF-AA increased the survival of endogenous OPCs and OLs, and promoted the proliferation of OPCs and their differentiation into OLs. Moreover, PDGF-AA also protected motor neurons from death in the injured spinal cord. These results indicated that PDGF-AA administration may be an effective treatment for SCI.

## Introduction

Spinal cord injury (SCI) is a devastating traumatic neurological disorder, resulting in permanent neurological deficits with limited prospects for spontaneous recovery (Quencer and Bunge, 1996; Ahuja et al., 2017). Recent statistical data showed that the estimated annual incidence of SCI was 54 cases per 1 million people in the United States in 2012 (Jain et al., 2015), an injury for which there are currently no effective treatments. SCI results in the death of oligodendrocytes (OLs) and neurons, leading to demyelination which further impairs neurological function (Liu et al., 1997; McTigue and Tripathi, 2008). Different strategies to promote endogenous repair and replace lost cells have been investigated for SCI in recent years (Thuret et al., 2006; Mothe and Tator, 2012; Silva et al., 2014; Assinck et al., 2017).

A previous study from our group reported that the co-transplantation of Schwann cells (SCs) and oligodendrocyte progenitor cells (OPCs) promoted the survival, proliferation, and migration of transplanted OPCs *in vivo*, and improved neurological recovery following SCI (Hu et al., 2013). A subsequent *in vitro* study found that SCs promote the proliferation and migration of OPCs by secreting platelet-derived growth factor (PDGF)-AA and fibroblast growth factor (FGF)-2 (Chen et al., 2015); moreover, we confirmed in an earlier study that PDGF-AA stimulated the proliferation of OPCs and their differentiation into OLs (Hu et al., 2008). Next, we showed that the transplanted PDGF-AA-overexpressing OPCs promoted tissue repair and recovery of neurological function in a rat model of SCI (Yao et al., 2017). However, it remains unclear whether treatment with PDGF-AA also affects endogenous OLs or even neurons, thus promoting further functional recovery after SCI.

In the present study, we evaluated the therapeutic potential of PDGF-AA administration by subcutaneous administration immediately after SCI. We demonstrated that injection of PDGF-AA promoted the proliferation of endogenous OPCs and their differentiation into OLs, increased the survival of OPC, OLs and motor neurons in the injured rat spinal cord, resulting in increased tissue sparing, myelination, and functional recovery post-SCI.

## Materials and Methods

### Animals

Adult (2 months old) female Sprague-Dawley rats (weighing 200–220 g) were used. All procedures were approved by the Animal Care and Use Committees of Bengbu Medical College, and were in accordance with the Guide for the Care and Use of Laboratory Animals (National Research Council, 1996) and the Guidelines and Policies for Rodent Survival Surgery, provided by the Animal Care and Use Committees of Bengbu Medical College. In total 32 rats were used in the study.

### Contusive SCI and PDGF-AA Administration

Contusive SCI was performed with the weight-drop method using a New York University Impactor (Gruner, 1992; Hu et al., 2013). Briefly, rats were anesthetized with pentobarbital (50 mg/kg) dissolved in phosphate buffered saline (PBS), injected intraperitoneally. Rats then received a laminectomy at the T10 level, and the dorsal surface of the spinal cord was subjected to a weight-drop impact of 10 g, dropped from a height of 12.5 mm. After SCI, rats were randomly assigned to the control-PBS or PDGF-AA injection group. PBS or human PDGF-AA (Invitrogen, Carlsbad, CA, United States; 300 μg/kg body weight each time based on our pretest) was subcutaneously injected into the back skin of rats at 30 min and every second day post-SCI for 2 weeks. The muscle and skin were sutured layer by layer and then sterilized with iodine. Rats were placed in a temperature- and humidity-controlled chamber, and post-operative monitoring included manual bladder emptying three times a day until reflexive bladder control was re-established. Moreover, rats received an analgesic agent (buprenorphine, 0.3 mg/kg) twice a day for 3 days to alleviate pain. To prevent infection, animals were provided with chloramphenicol (50–75 mg/kg) daily via the drinking water. Subsequently, animals were sacrificed at 2 or 6 weeks post-SCI.

### Behavioral Assessment

Open-field locomotor testing was carried out by two trained investigators using the 21-point Basso, Bresnahan, and Beattie (BBB) locomotor scale (Basso et al., 1995) once a week post-injury to assess hindlimb locomotor recovery, including joint movement, stepping ability, coordination, and trunk stability.

### Tissue Preparation

At predetermined time points (2 and 6 weeks post-injury), rats were anesthetized with 60 mg/kg pentobarbital and transcardially perfused with 4% paraformaldehyde (PFA) in 0.01 M PBS, pH 7.4. Spinal cord segments containing the injury site were removed, post-fixed in the same fixative overnight at 4°C, and cryoprotected in 30% sucrose (Sigma-Aldrich, St. Louis, MO, United States) buffer for 5–7 days. A 2 cm length of the spinal cord, centered at the injection or injury site, was dissected and embedded in HistoPrep (Fisher Scientific, Pittsburgh, PA, United States) on dry ice. After the spinal cords were mounted in blocks, serial 20 μm-thick sections through the entire injury site were cut on a cryostat. All spinal cords from each group (*n* = 8 rats/group) were cut as transverse sections. Sections were mounted on gelatin-coated slides (Fisher Scientific, Waltham, MA, United States), and stored at -70°C.

### Histological Analysis

Two sets of transverse sections (each set containing serial sections spaced 500 μm apart) from rat spinal cord at 6 weeks post-SCI were stained with cresyl violet-eosin (Sigma) for lesion area assessment (*n* = 8/group) and Luxol Fast Blue (LFB; Sigma) for white matter sparing analysis (*n* = 8/group), as previously described (Hu et al., 2013). The total area of cavitation and LFB-positive myelinated areas in axial sections at the injury epicenter and at 1, 2, and 4 mm rostral and caudal to the injury epicenter were outlined and measured using the Neurolucida System (MicroBrightField, Colchester, VT, United States) connected to a BX60 microscope (Olympus, Tokyo, Japan), and are expressed as a percentage of the total stained area. The lesion center was defined as the section containing the least amount of spared white matter.

### Immunohistochemistry

Cryostat sections were stained using standard immunohistochemistry techniques. Frozen sections collected on slides were air-dried at room temperature for 10 min and washed with PBS for 10 min, then blocked with Tris-buffered saline containing 10% donkey serum and 0.3% Triton X-100 for 1 h at room temperature. Primary antibodies in the same blocking solution were applied overnight at 4°C. The primary antibodies used were as follows: rabbit anti-caspase-3 antibody (1:200; Abcam, Cambridge, MA, United States), mouse anti-NG2 (1:200; Abcam), mouse anti-beta III tubulin (1:200; Abcam), and mouse anti-CNPase (CNP; 1:200; Abcam). The slides were then washed three times in PBS and incubated with fluorescein isothiocyanate (FITC)- or rhodamine-conjugated donkey anti-rabbit or mouse IgG (all at 1:200; Jackson Laboratores, West Grove, PA, United States) for 1 h at 37°C. Slides were washed three times with PBS and mounted with Gel/Mount containing Hoechst 33342 to counterstain the nuclei. Images were acquired with a Zeiss Axio Observer fluorescence microscope. Control samples were prepared by omitting the primary antibody. The spinal cord cross-sections from six rats per timepoint were used for evaluation. Five complete sections per animal were analyzed in a blinded fashion, and cell numbers were calculated as the number of cells in a set of five slides, from rostral to caudal, containing the injury epicenter.

### Bromodeoxyuridine (BrdU) Incorporation Assay

To assess cell proliferation after PDGF-AA administration, 3 days prior to the end of the 2-week post-SCI timepoint, randomly selected rats (*n* = 8 rats/group) were given 10 intraperitoneal injections of BrdU (50 mg/kg/injection; three times daily for 3 days, and one injection on the last day). Rats were sacrificed 2 h after the last injection. The animals were perfused, their spinal cords were dissected, and the tissue was sectioned. One set of serial sections was randomly selected for the BrdU incorporation assay. Fixed sections were treated with 1N HCl for 40 min at 37°C to denature the DNA. The rabbit anti-BrdU antibody (1:100; Sigma-Aldrich) and the mouse anti-O4 (1:100; Chemicon, Temecula, CA, United States), or CNP (1:200; Abcam) antibodies were applied overnight at 4°C, followed by FITC-conjugated donkey anti-rabbit IgG (1:200; Jackson Labs) and rhodamine- conjugated donkey anti-mouse IgM (1:200; Jackson Labs) or IgG (1:200; Jackson Labs) as secondary antibodies at room temperature for 2 h. Slides were washed three times with PBS and mounted with Gel/Mount. Images were acquired with a Zeiss Axio Observer fluorescence microscope. At least five randomly selected fields in each section with a total of more than 500 O4+ or CNP+ cells were counted. The percentage of O4+/BrdU+ or CNP+/BrdU+ cells out of the total number of O4+ or CNP+ cells was determined.

### Statistical Analysis

Data are presented as the mean ± standard deviation (SD). A one-way analysis of variance (ANOVA) with a Tukey’s or Fisher’s *post hoc* test was used to evaluate mean differences. BBB scores were analyzed using a repeated measures ANOVA, followed by a Tukey’s pairwise comparison at each timepoint. Other data were analyzed using a non-parametric Kruskal–Wallis ANOVA, followed by individual Mann–Whitney *U*-tests. A *P*-value < 0.05 was considered statistically significant. Data were analyzed using SPSS v.14.0 software (SPSS, Inc., Chicago, IL, United States).

## Results

### Treatment With PDGF-AA Promotes Functional Recovery After SCI

**FIGURE 1:**
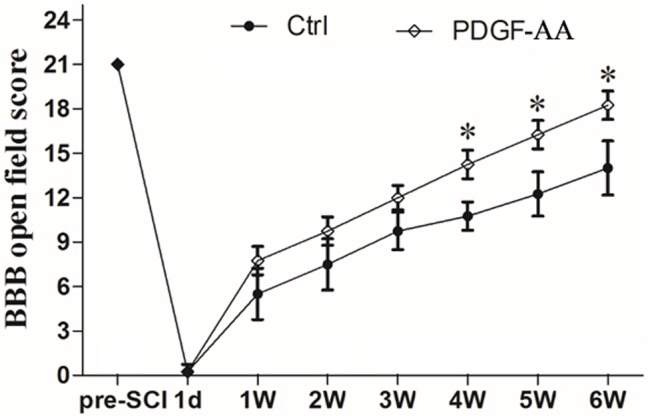
Treatment with PDGF-AA improves locomotor functional recovery after SCI. Spinal cord-injured rats that received treatment with PDGF-AA showed greater improvement in locomotor BBB scores at 4–6 weeks post-SCI, as compared to controls (*n* = 8). ^∗^*P* < 0.05.

In order to examine whether subcutaneous PDGF-AA injections could promote functional recovery after SCI, BBB scoring was performed. One day after SCI, all injured rats were paraplegic with no observable hind limb movement. In the first 3 weeks after injection of PDGF-AA, there was no difference between the control and PDGF-AA-treated groups in terms of BBB score (Figure 1). However, with continued recovery, rats that were administered PDGF-AA showed a significant improvement in their BBB score, as compared to the control group, at 4–6 weeks post-injection (*P* < 0.05, *n* = 8; Figure 1). Thus, the PDGF-AA injection regime resulted in improved functional recovery.

### Treatment With PDGF-AA Increases Tissue Sparing in the Injured Spinal Cord

**FIGURE 2:**
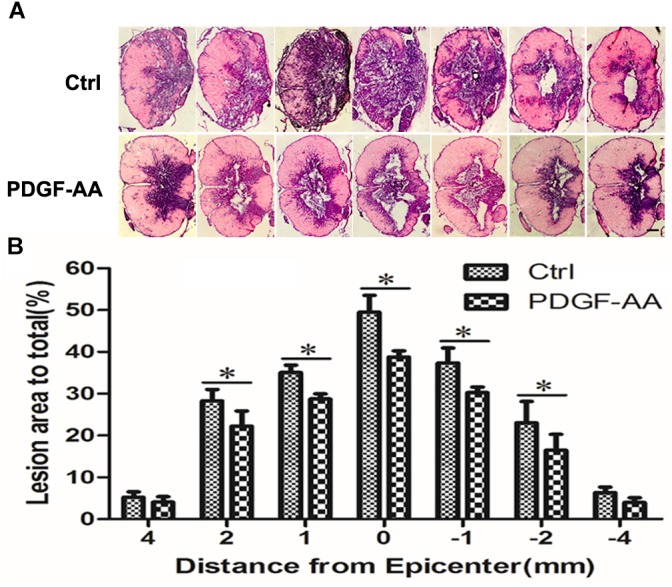
Tissue sparing in the spinal cord following SCI. **(A)** Representative images of sections stained with cresyl violet-eosin, showing the extent of tissue sparing at the lesion center 6 weeks post-SCI in the PDGF-AA group versus the control group. **(B)** Quantitative analysis of the total area of cavitation in axial sections at the injury epicenter and at 1, 2, and 4 mm rostral and caudal to the injury epicenter. The caviated area was significantly different between the two groups (*n* = 8). ^∗^*P* < 0.05. Scale bar: 200 μm.

H&E staining was performed in order to examine histological morphology of the injury site at 6 weeks post-SCI. Consistent with the locomotion evaluation, the PDGF-AA-treated group showed less tissue damage, with a smaller lesion area compared to the control group (*P* < 0.05, *n* = 8; Figures 2A,B), indicating that treatment with PDGF-AA increased tissue sparing in the injured spinal cord, and ameliorated the pathological morphology of the lesion area.

### Treatment With PDGF-AA Increases Myelination in the Injured Spinal Cord

**FIGURE 3:**
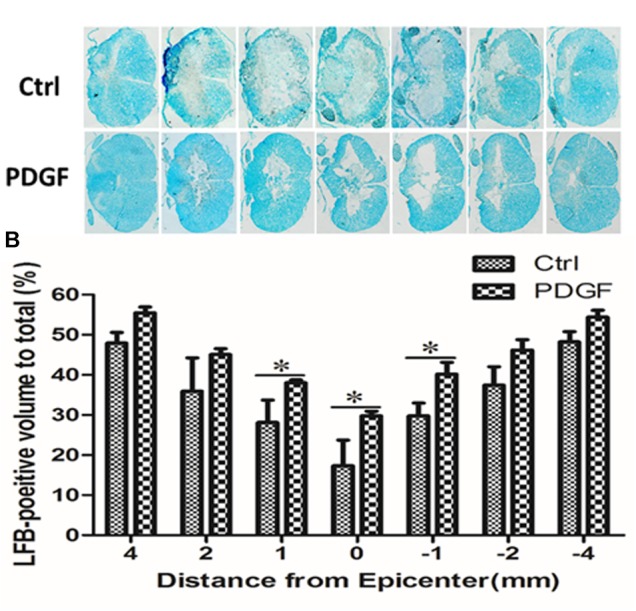
Quantitative analysis of residual myelination in the injured spinal cord 6 weeks post-SCI. **(A)** LFB-stained spinal cord cross-sections from the injury epicenter (0) and at 1, 2, and 4 mm rostral (+) and caudal (–) to the epicenter in the PDGF-AA-treated group and the control group. **(B)** Quantification of the LFB-positive myelinated areas between the two groups at various distances from the injury epicenter. Data represent the mean ± SD of three independent experiments (*n* = 8). ^∗^*P* < 0.05.

To investigate whether administration of PDGF-AA improved the preservation of existing myelin and/or promotes re-myelination, LFB staining was performed to assess the extent of myelination at the injury center 6 weeks post-PDGF-AA injection. We observed that the area of LFB staining at the injury center was larger in rats treated with PDGF-AA, compared to the control group that did not receive PDGF-AA (*P* < 0.05, *n* = 8; Figures 3A,B).

### Treatment With PDGF-AA Promotes the Survival of OLs and OPCs in the Injured Spinal Cord

**FIGURE 4:**
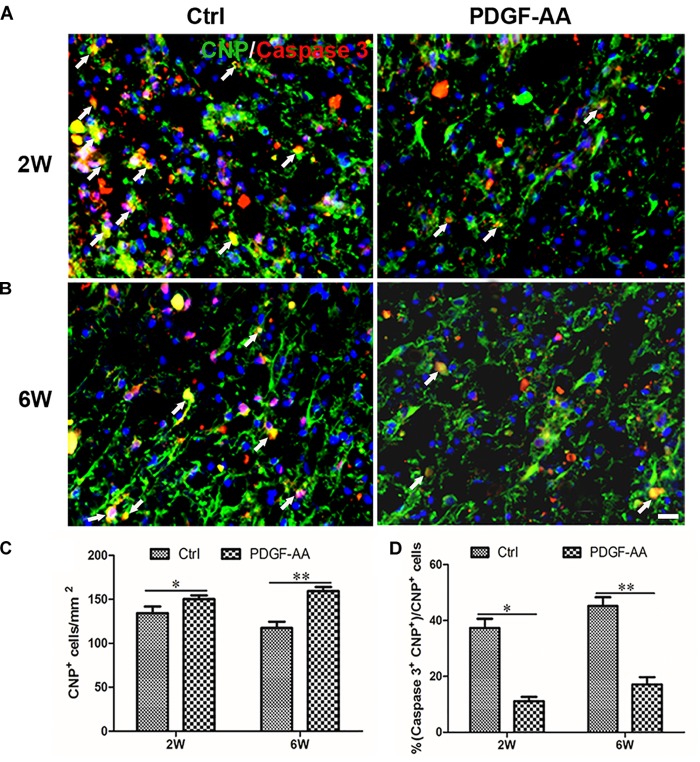
Proliferation of OPCs in the injured spinal cord post-SCI, as determined by BrdU incorporation. **(A,B)** Representative photomicrographs showing BrdU+ cells (green) co-localized with O4+OPCs (red) in the circumambience of the injured spinal cord of rats at 2 and 6 weeks post-SCI. **(C,D)** Quantitative analysis of OPC proliferation, demonstrating an increase in both the number and percentage of BrdU+OPCs in rats that received PDGF-AA versus controls. Data represent the mean ± SD (*n* = 8). ^∗^*P* < 0.05; ^∗∗^*P* < 0.01. Scale bar = 20 μm.

**FIGURE 5:**
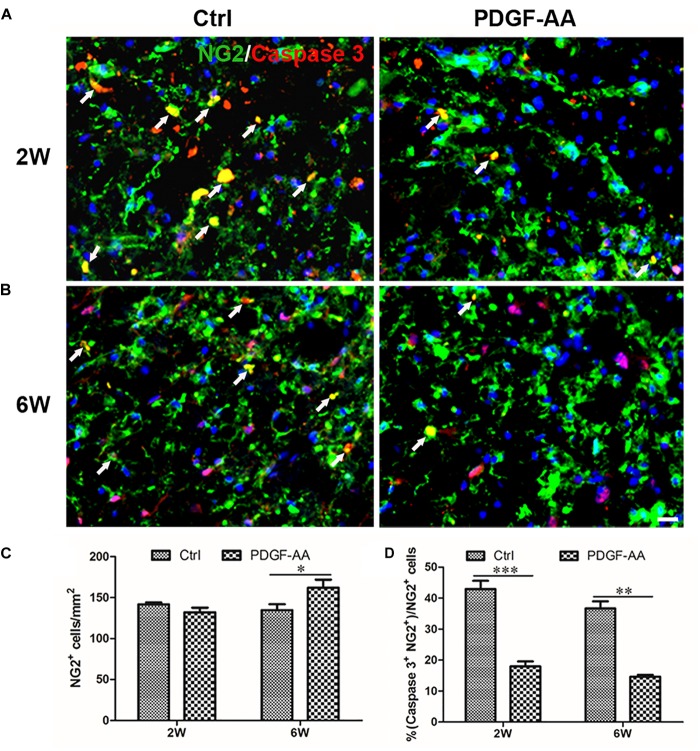
Differentiation of OLs from proliferated OPCs in the injured spinal cord after SCI, as determined by BrdU incorporation. **(A,B)** Representative photomicrographs showing BrdU+cells (green) co-localized with CNP+OLs (red) in the circumambience of the injured spinal cord of rats at 2 and 6 weeks post-SCI. **(C,D)** Quantitative analysis of BrdU+CNP+OLs showing that both the number and percentage of BrdU+OLs were higher in rats that received PDGF-AA versus controls. Data represent the mean ± SD (*n* = 8). ^∗^*P* < 0.05; ^∗∗^*P* < 0.01; ^∗∗∗^*P* < 0.001. Scale bar = 20 μm.

Activated caspase-3 staining revealed that at both 2 and 6 weeks there were more CNP+ OLs (112.75 ± 3.11 versus 98 ± 2.83% at 2 weeks, *P* < 0.05, *n* = 8, Figures 4A,C; 121 ± 0.71 versus 88 ± 5.48% at 6 weeks, *P* < 0.05, *n* = 8, Figures 4B,C) and fewer caspase-3+/CNP+ OLs (11.12 ± 1.54 versus 37.33 ± 3.31% at 2 weeks, *P* < 0.05, *n* = 8, Figures 4A,D; 17.14 ± 2.59 versus 45.23 ± 3.07% at 6 weeks, P < 0.05, *n* = 8, Figures 4B,D) at the periphery of the injury center in rats treated with PDGF-AA, as compared to control rats that did not receive PDGF-AA. Similarly, more NG2+ OPCs (121.5 ± 7.40 versus 101.12 ± 5.39% at 6 weeks, *P* < 0.05, *n* = 8, Figures 5B,C) and fewer caspase-3+/NG2+ OPCs (17.97 ± 1.58 versus 42.95 ± 2.66% at 2 weeks, *P* < 0.05, *n* = 8, Figures 5A,D; 14.60 ± 0.62 versus 36.67 ± 2.32% at 6 weeks, *P* < 0.05, *n* = 8, Figures 5B,D) at the periphery of the injury center were observed in rats treated with PDGF-AA as compared to control rats that did not receive PDGF-AA.

### Treatment With PDGF-AA Promotes the Proliferation of OPCs and Their Differentiation Into OLs in the Injured Spinal Cord

**FIGURE 6:**
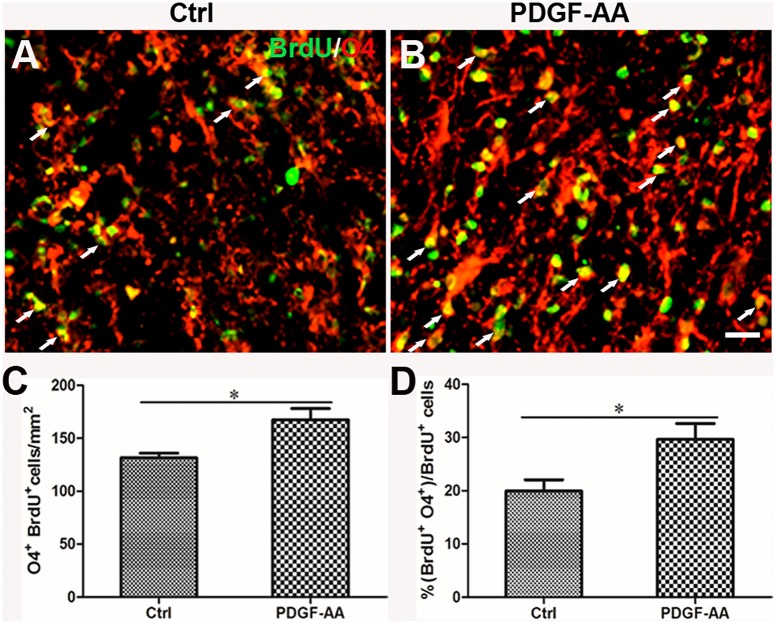
Survival of OLs in the injured spinal cord after SCI, as determined by activated caspase-3 staining. **(A,B)** Representative photomicrographs showing caspase-3+cells (red) co-localized with CNP+OLs (green) in the circumambience of the injured spinal cord of rats at 2 weeks post-SCI. **(C,D)** Quantitative analysis of OL survival showing that both the number and percentage of caspase-3+OLs were higher in rats that received PDGF-AA versus controls. Data represent the mean ± SD (*n* = 8). ^∗^*P* < 0.05. Scale bar = 20 μm.

**FIGURE 7:**
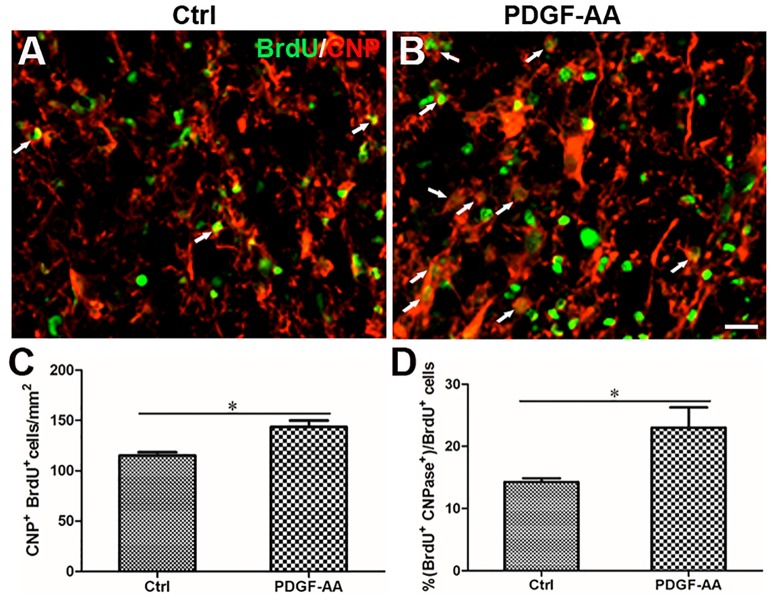
Survival of OPCs in the injured spinal cord after SCI, as determined by activated caspase-3 staining. **(A,B)** Representative photomicrographs showing caspase-3+cells (red) co-localized with NG2+OPCs (green) in the circumambience of the injured spinal cord of rats at 2 weeks post-SCI. **(C,D)** Quantitative analysis of OPC survival showed that both the number and percentage of caspase-3+ OPCs were higher in rats that received PDGF-AA versus controls. Data represent the mean ± SD (*n* = 8). ^∗^*P* < 0.05. Scale bar = 20 μm.

A BrdU incorporation assay at 2 weeks post-SCI revealed an increase in the number of both BrdU+/O4+ OPCs (167.33 ± 9.31 versus 131.56 ± 3.50%, *P* < 0.05, *n* = 8, Figures 6A–C) and BrdU+/CNP+ OLs (143.67 ± 5.45 versus 115.11 ± 2.74%, *P* < 0.05, *n* = 8, Figures 7A–C) at the injury center in rats treated with PDGF-AA as compared to control rats that did not receive PDGF-AA. Similarly, the percentages of BrdU+ cells among both O4+ cells (29.52 ± 2.76 versus 19.95 ± 1.71%, *P* < 0.05, *n* = 8, Figures 6A,B,D) and CNP+ cells (23.00 ± 2.82 versus 14.26 ± 0.47%, *P* < 0.05, *n* = 8, Figures 7A,B,D) at the injury center were greater in rats treated with PDGF-AA as compared to controls that did not receive PDGF-AA. These data confirmed that treatment with PDGF-AA promoted the proliferation of endogenous OPCs and their differentiation into OLs in the injured spinal cord post-SCI.

### Treatment With PDGF-AA Promotes the Survival of Motor Neurons in the Ventral Horn Following SCI

**FIGURE 8:**
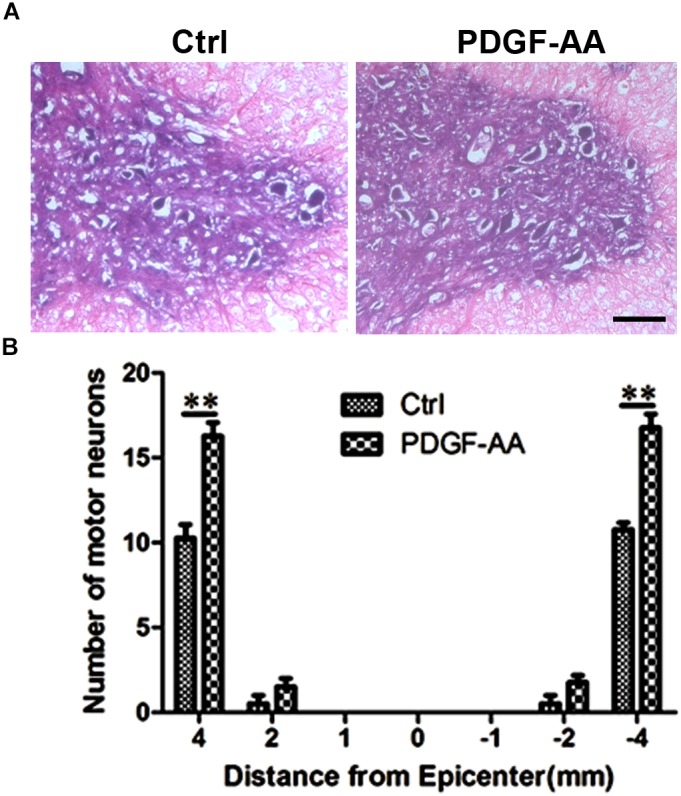
Motor neuron survival in the ventral horn of the spinal cord 6 weeks post-SCI. **(A)** Representative images of the ventral horn 4 mm rostral to the injury epicenter in the control and PDGF-AA-treated groups. **(B)** Comparison of the numbers of ventral horn neurons in different groups at various distances from the injury epicenter (0) and at 1, 2, and 4 mm rostral (+) and caudal (–) to the epicenter. Data represent the mean ± SD of three independent experiments (*n* = 8). ^∗∗^*P* < 0.01. Scale bar = 200 μm.

To determine the effect of PDGF-AA administration on motor neuronal survival, the number of ventral horn motor neurons at the injury epicenter and at 1, 2, and 4 mm rostral and caudal to the epicenter were counted 6 weeks post-SCI. An increase in the number of residual motor neurons was observed in the ventral horn 4 mm rostral and caudal to the lesion epicenter in the PDGF-AA-treated group, as compared to the control group (*P* < 0.01, *n* = 8, Figure 8).

## Discussion

We previously found that co-transplantation of SCs enhanced functional recovery in a rat model of SCI (Hu et al., 2013) via secretion of PDGF-AA and FGF-2 (Chen et al., 2015). Subsequently, we showed that the transplantation of PDGF-AA-overexpressing OPCs promoted tissue repair and recovery of neurological function after SCI (Yao et al., 2017). In the present study, the spinal cord injured rats received subcutaneous administration of PDGF-AA to investigate whether it effects endogenous OLs and other neural cells to lead to a therapeutic effect.

In this study, we found no difference in BBB score during the first 3 weeks post-SCI in rats with or without PDGF-AA administration. However, at 4–6 weeks post-SCI, we observed a significant improvement in the BBB score in rats that received PDGF-AA injections, as compared to control rats that did not receive PDGF-AA injections. This behavioral finding was consistent with our previous results of OPC/SC co-transplantation (Hu et al., 2013), as well as results from the transplantation of PDGF-AA-overexpressing OPCs (Yao et al., 2017). This result suggested that PDGF-AA administration was able to promote functional locomotor recovery after SCI in rats.

We next evaluated whether PDGF-AA injection promoted tissue repair in the injured spinal cord. We observed a significant reduction in the spinal cord lesion area in rats that received PDGF-AA injections compared to control rats, indicating that PDGF-AA injection enhanced tissue repair following SCI, which explained the functional improvement observed in these animals.

Preservation of existing myelin and re-myelination is the basis of functional recovery following SCI (Keirstead et al., 2005; Xu and Onifer, 2009). In our study, there was more residual myelin in rats that received PDGF-AA injection as compared to control rats at 6 weeks post-SCI, indicating that treatment with PDGF-AA preserved residual myelin and promoted the re-myelination of demyelinated axons in the injured spinal cord.

Spinal cord injury leads to the death of a massive number of cells, including neurons and OLs that myelinate the axons of surviving neurons (Casha et al., 2001; Beattie et al., 2002; Stenudd et al., 2015). It has been reported that PDGF, in synergy with bFGF, regulates the proliferative response of adult OPCs (Lachapelle et al., 2002; Frost et al., 2003; Karimi-Abdolrezaee et al., 2012). Our previous study also confirmed that overexpression of PDGF-AA promoted the proliferation and survival of transplanted OPCs (Yao et al., 2017), which resulted in increased myelination and tissue repair, leading to improved functional recovery. To investigate the mechanism by which PDGF-AA treatment promoted functional recovery and tissue repair following SCI, we examined the effect of PDGF-AA treatment on the survival and proliferation of endogenous OLs around the injury site in the spinal cord following SCI. Activated caspase-3 labeling and BrdU incorporation revealed that PDGF-AA administration protected both OPCs and OLs from apoptosis, and promoted the proliferation of OPCs and their differentiation into OLs after SCI. These results suggested that PDGF-AA treatment increased the number of myelin-forming cells at the injury site post-SCI, which led to further myelin repair and functional recovery following SCI.

The death of neurons is another key factor that results in the loss of function after SCI (Siddiqui et al., 2015). It has been reported that PDGF-AA suppresses the Ca^2+^ overload induced by H_2_O_2_ in mouse cortical neuron primary cultures (Zheng et al., 2013), and protects them from H_2_O_2_-induced oxidative stress (Zheng et al., 2010). Moreover, Cheng and Mattson (1995) found that pretreatment of rat and mouse hippocampal neurons with PDGF-AA resulted in a highly significant attenuation of glucose deprivation- and FeSO_4_-induced neuronal degeneration. However, it remained unclear whether PDGF-AA injection affected the survival of neurons in the injured spinal cord after SCI. In this study, we observed an increase in the number of residual motor neurons in the ventral horn rostral and caudal to the lesion epicenter in PDGF-AA-treated rats. This result suggested that treatment with PDGF-AA promoted the survival of neurons following SCI. Together with the observed effect of PDGF-AA treatment on the survival of endogenous OPCs and OLs, and the proliferation of OPCs and their differentiation into OLs, our results clarified why PDGF-AA treatment promoted the repair of injured spinal cord tissue, resulting in improved functional recovery after SCI.

## Conclusion

Our results demonstrated that PDGF-AA treatment promoted the survival of OLs and neurons and increased the proliferation of endogenous OPCs and their differentiation into OLs post-SCI, which increased the amount of myelination and tissue repair in the injured spinal cord, leading to improved recovery of neurological function. These results suggested that treatment with PDGF-AA may be a potential strategy to promote recovery following SCI.

## Author Contributions

J-GH and H-ZL designed the experiments and edited the manuscript. X-YG, F-XD, and JC performed the experiments, analyzed the data, and wrote the manuscript. YW, RW, and Z-QJ interpreted the data and prepared the figures. LS, QQ, JX, and A-YZ performed the experiments and analyzed the data.

## Conflict of Interest Statement

The authors declare that the research was conducted in the absence of any commercial or financial relationships that could be construed as a potential conflict of interest.
